# Histamine *N*-Methyltransferase in the Brain

**DOI:** 10.3390/ijms20030737

**Published:** 2019-02-10

**Authors:** Takeo Yoshikawa, Tadaho Nakamura, Kazuhiko Yanai

**Affiliations:** 1 Department of Pharmacology, Tohoku University Graduate School of Medicine, 2-1, Seiryo-machi, Aoba-ku, Sendai 980-8575, Japan; tadaho@med.tohoku.ac.jp (T.N.); yanai@med.tohoku.ac.jp (K.Y.); 2 Division of Pharmacology, Faculty of Medicine, Tohoku Medical and Pharmaceutical University, 1-15-1, Fukumuro, Miyagino-ku, Sendai 983-8536, Japan

**Keywords:** diamine oxidase, histamine, histamine H_3_ receptor, histamine *N*-methyltransferase

## Abstract

Brain histamine is a neurotransmitter and regulates diverse physiological functions. Previous studies have shown the involvement of histamine depletion in several neurological disorders, indicating the importance of drug development targeting the brain histamine system. Histamine *N*-methyltransferase (HNMT) is a histamine-metabolising enzyme expressed in the brain. Although pharmacological studies using HNMT inhibitors have been conducted to reveal the direct involvement of HNMT in brain functions, HNMT inhibitors with high specificity and sufficient blood–brain barrier permeability have not been available until now. Recently, we have phenotyped *Hnmt*-deficient mice to elucidate the importance of HNMT in the central nervous system. *Hnmt* disruption resulted in a robust increase in brain histamine concentration, demonstrating the essential role of HNMT in the brain histamine system. Clinical studies have suggested that single nucleotide polymorphisms of the human *HNMT* gene are associated with several brain disorders such as Parkinson’s disease and attention deficit hyperactivity disorder. Postmortem studies also have indicated that HNMT expression is altered in human brain diseases. These findings emphasise that an increase in brain histamine levels by novel HNMT inhibitors could contribute to the improvement of brain disorders.

## 1. Introduction

Histamine (2-[4-imidazolyl]ethylamine) was discovered by Sir Henry Hallet Dale and Sir Patrick Playfair Laidlaw in 1910 [1]. Histamine mediates a wide variety of events such as allergic reactions, gastric acid secretion, and smooth muscle contraction through interaction with four histamine receptor subtypes (H_1_R–H_4_R). Pharmacological research targeting histamine action in peripheral organs has led to the development of drugs such as H_1_R antagonists for allergic diseases and H_2_R antagonists for gastric ulcers. This amine also acts as a neurotransmitter in the brain [2]. Histaminergic neurons are located in the tuberomammillary nucleus of the hypothalamus and project their axons into various brain regions including the cerebral cortex, hypothalamus, basal ganglia, and amygdala [3]. The number of histamine-producing neurons in the human brain is estimated to be approximately 64,000. Brain histamine regulates diverse physiological functions such as sleep–wake cycles, stress response, appetite, and memory. Extensive investigations have shown the pathophysiological involvement of the histaminergic nervous system in various neuropsychiatric disorders. A loss-of-function mutation of the *histidine decarboxylase* (*HDC*) gene (EC 4.1.1.22), the essential enzyme for histamine production, is associated with Tourette’s syndrome [4]. Pathological changes in histamine neurons are involved in cognitive impairment [5,6,7]. The reduction in histamine concentration in the cerebrospinal fluid was observed in narcolepsy patients [8,9,10]. H_1_R binding potential was found to be decreased in patients with depression and schizophrenia (SCZ) via positron emission tomography [11,12]. This evidence suggests that the dysfunction of the histaminergic nervous system could play a causative role in various neurological disorders and that the increase in brain histamine might have therapeutic potential.

Over the past few decades, H_3_R receptors in the brain have attracted attention as therapeutic targets to modulate the histamine system. This receptor identified by Arrang et al. in 1983 is a Gi/o protein-coupled receptor and negatively regulates histamine release as a presynaptic autoreceptor at nerve end terminals [2,13]. H_3_R inverse agonists, which could induce histamine release, were developed by many pharmaceutical companies and were examined for their therapeutic effects on animal models for neurological disorders and also on patients suffering from brain diseases [14,15]. Although the beneficial effect of most H_3_R inverse agonists is inconclusive, pitolisant, an H_3_R antagonist developed by Schwartz et al. [16], was approved for the treatment of narcolepsy by the European Medical Agency in 2016 [17], emphasising the involvement of histamine concentration in brain functions.

Neurotransmitter clearance is very important for maintaining normal neurotransmitter concentration. The neurotransmitters released into extracellular spaces are cleared by transporters and /or enzymes in the adjacent neurons or astrocytes to avoid excessive neuronal activation. Dysfunctional neurotransmitter clearance plays a causative role in various neurological disorders, including SCZ and depression. Indeed, various drugs, such as tricyclic antidepressants, serotonin norepinephrine re-uptake inhibitors, and monoamine oxidase inhibitors, block neurotransmitter clearance and exert their therapeutic actions in patients suffering from brain diseases. This evidence supports that histamine clearance machinery could be a therapeutic target for developing novel drugs that improve brain functions. However, the mechanism of brain histamine clearance had not been clarified.

**Figure 1:**
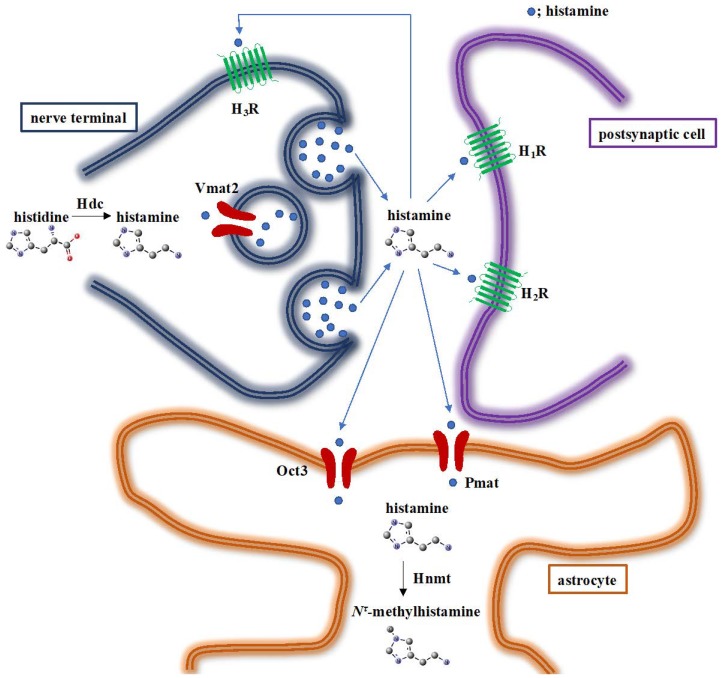
Neurotransmission and termination of histaminergic nervous system. Histidine decarboxylase (Hdc) synthesises histamine from histidine. Histamine is stored in synaptic vesicles via vesicular monoamine transporter 2 (Vmat2). Upon stimulation, histamine is released to extraneuronal spaces. Histamine exerts its effects through interactions with postsynaptic histamine h1 receptor (H_1_R) and H_2_R, and presynaptic H_3_R. Extracellular histamine is transported via organic cation transporter 3 (Oct3) and plasma membrane monoamine transporter (Pmat). Finally, histamine is metabolised by histamine *N*-methyltransferase (Hnmt) [19,32]. Although previous studies have shown the importance of astrocytes for histamine clearance in the CNS [18], several reports suggest the involvement of neurons in histamine clearance [33,34].

Previous studies using rodents showed that astrocytes play an important role in histamine clearance [18]. We recently investigated the molecular mechanism of histamine clearance using primary human astrocytes [19]. Our in vitro experiments showed that human astrocytes transport histamine dominantly through plasma membrane monoamine transporter (PMAT) and also through organic cation transporter 3 (OCT3). Next, the histamine transported into the cytosol is metabolised by histamine *N*-methyltransferase (HNMT) (EC 2.1.1.8) (Figure 1). PMAT and OCT3, which are polyspecific transporters, transport various monoamines, including serotonin, dopamine, norepinephrine, and histamine [20]. PMAT, which was identified in 2004, is widely distributed in the brain [21,22]. Several reports suggested that PMAT plays a role in serotonin clearance [23,24], and that mutations in the human *PMAT* genes coupled with low transport activity are related to autism spectrum disorders [25], indicating the involvement of PMAT in brain monoamine concentration. However, *Pmat* deficiency in mice does not greatly affect brain histamine concentration under non-stressful conditions (our unpublished observation) nor induces behavioural abnormalities [26]. OCT3 is expressed in different brain regions including the cerebral cortex, hippocampus, and cerebellum [27]. The contribution of OCT3 to serotonin and dopamine concentrations has already been reported [28,29]. Zhu et al. examined the importance of OCT3 in brain histamine concentration [30]. They showed that OCT3 is not involved in brain histamine concentration in normal conditions, whereas histamine content in the brain cortex is elevated in *Oct3*-deficient mice after cerebral ischemia. These results might indicate the minor contribution of these transporters to brain histamine concentration, although further studies are essential to examine the importance of PMAT and OCT3 in histamine clearance. In contrast, our recent study using *Hnmt* knockout mice clearly showed that Hnmt plays a predominant role in brain histamine concentration and the regulation of the histaminergic nervous system [31]. In this article, we focus on HNMT function in the central nervous system (CNS).

## 2. Histamine-Metabolising Enzymes

There are two different enzymes for the inactivation of histamine: diamine oxidase (DAO) (EC 1.4.3.22) and HNMT. DAO, also known as histaminase, is the product of the *AOC1* gene. DAO acts as a homodimeric protein to oxidatively deaminate various amines including histamine, putrescine, and spermidine [35,36]. DAO is highly expressed in the digestive tract. The K_m_ (Michaelis constant) value of human intestinal DAO to histamine was calculated as 19 µM [37]. DAO plays a role in detoxification of dietary histamine to reduce histamine uptake through enterocytes. Thus, impaired DAO activity results in the increase of histamine absorption and the elevation of blood histamine concentration. Although DAO is also highly expressed in the kidneys and placenta, DAO expression in the CNS is low or absent [38], indicating that DAO metabolises histamine in the peripheral organs but not the CNS.

**Figure 2:**
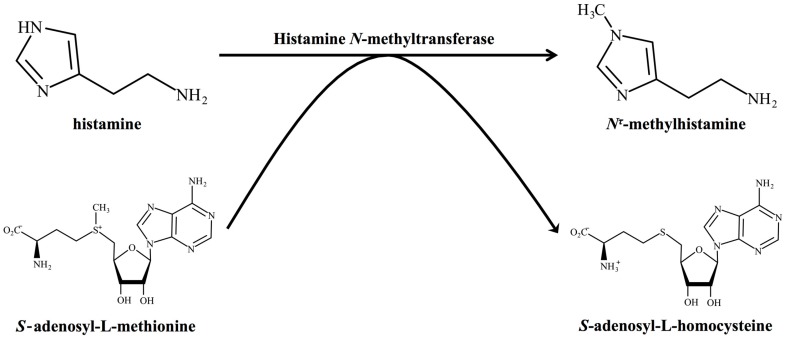
Enzymatic activity of histamine *N*-methyltransferase. Histamine *N*-methyltransferase catalyses the transfer of a methyl group from S-adenosyl-l-methionine to histamine, yielding *N^τ^*-methylhistamine and S-adenosyl-l-homocysteine.

HNMT is an enzyme catalysing the transfer of a methyl group from S-adenosyl-l-methionine (SAM) to histamine, yielding *N^τ^*-methylhistamine and S-adenosyl-l-homocysteine (Figure 2). The human *HNMT* gene was cloned by Girard et al. in 1994 and encodes a 33 kDa protein consisting of 292 amino acids [39] (Table 1). Although HNMT is widely observed in vertebrates including humans, rodents, birds, lizards, and amphibians, the expression of HNMT has not been confirmed in invertebrates and plants. In mammals, HNMT is widely expressed in various organs including liver, kidney, and brain [40]; and methylated histamine metabolites are abundantly excreted in urine [41], suggesting the important role of HNMT in histamine metabolism. Brain HNMT activity in the CNS was first detected in the soluble supernatant fraction from guinea pig brain in 1959 [42]. Schayer and Reilly confirmed the existence of methylated histamine inactivated by HNMT in guinea pig brain [43]. Human HNMT activity has also been detected in the frontal, temporal, parietal, occipital, and cerebellar cortices [44]. The Human Protein Atlas project showed a high expression of HNMT in the cerebellum and medium expression in the cerebral cortex, hippocampus, and caudate [45]. The project also found that both neurons and glial cells express HNMT. An *in situ* hybridisation study in Allen Mouse Brain Atlas showed the highest mRNA expression of *HNMT* in the cortical subplate [46]. Northern blot analysis using mouse and rat brains revealed ubiquitous expression of *Hnmt* except in rat cerebellum and mouse striatum [47]. Immunohistochemical analysis using bovine brain revealed that several neurons including the oculomotor nucleus, red nucleus, facial nucleus, and dorsal vagal nucleus strongly express Hnmt [33]. However, the detailed distribution of brain HNMT in mice, rats, and humans is still unknown. Further immunohistochemical analysis using specific antibodies is essential to understanding detailed HNMT distribution in the CNS.

## 3. HNMT and Human Brain Diseases

The effects of genetic polymorphism of human *HNMT* on enzymatic activity was first reported by Preuss et al. in 1998 [40]. They showed that a C-to-T transition at nucleotide 314 (C314T) in exon 4 replaces threonine with isoleucine at codon 105 (Thr105Ile), leading to decreased enzymatic activity. Although amino acid residue 105 is distal from the substrate binding sites, the C314T polymorphism has a great impact on active site structure and dynamics, resulting in the increased K_m_ value of human HNMT for histamine and SAM by 1.3- and 1.8-fold, respectively [52,53]. Several groups examined the association of C314T polymorphism with Parkinson’s disease (PD) [54,55,56,57], and the meta-analysis of these four studies suggested a protective role of this polymorphism against the development of PD [58] (Table 2). Other genetic association studies suggested the involvement of the C314T substitution in SCZ [57], attention deficit hyperactivity disorder (ADHD) [59] and migraine [60] but not in Alzheimer’s disease (AD) [61], amyotrophic lateral sclerosis [62], multiple sclerosis [63], or restless legs syndrome [64]. The effect of the C314T polymorphism on alcoholism was inconclusive [65,66]. An A-to-G polymorphism at nucleotide 939 in the 3′ untranslated region of the human *HNMT* gene increases *HNMT* mRNA stability and increases HNMT protein, and enhances enzymatic activity [67]. An A939G polymorphism is related to several brain disorders such as myasthenia gravis and ADHD [59,68]. Recently, Heidari et al. reported that two novel mutations in the human *HNMT* gene (G179A and T632C) impairs its enzymatic activity, leading to intellectual disability [69,70].

Several postmortem studies examined the alteration of HNMT expression in neurological disorders. HNMT expression was increased at the inferior frontal gyrus in Huntington’s disease [71], at the substantia nigra and putamen in PD [72], and at the frontal cortex in Pick’s disease [73]. *HNMT* mRNA expression was also elevated in the prefrontal cortex of female AD patients [74]. On the other hand, HNMT expression was reduced at the anterior cingulated cortex in depression [75] and the frontal cortex in Down syndrome [73]. Although these results indicate the possible involvement of HNMT functions in neuropsychiatric disorders, it is still unknown whether alterations in HNMT activity exert the causative role in disease progression, play a compensatory function for impaired brain functions, or is a secondary outcome accompanied by primary pathological change. Therefore, these associations should be validated in larger studies and possibly by prospective clinical studies in the near future.

## 4. Pharmacological Analysis Using HNMT Inhibitors

**Figure 3:**
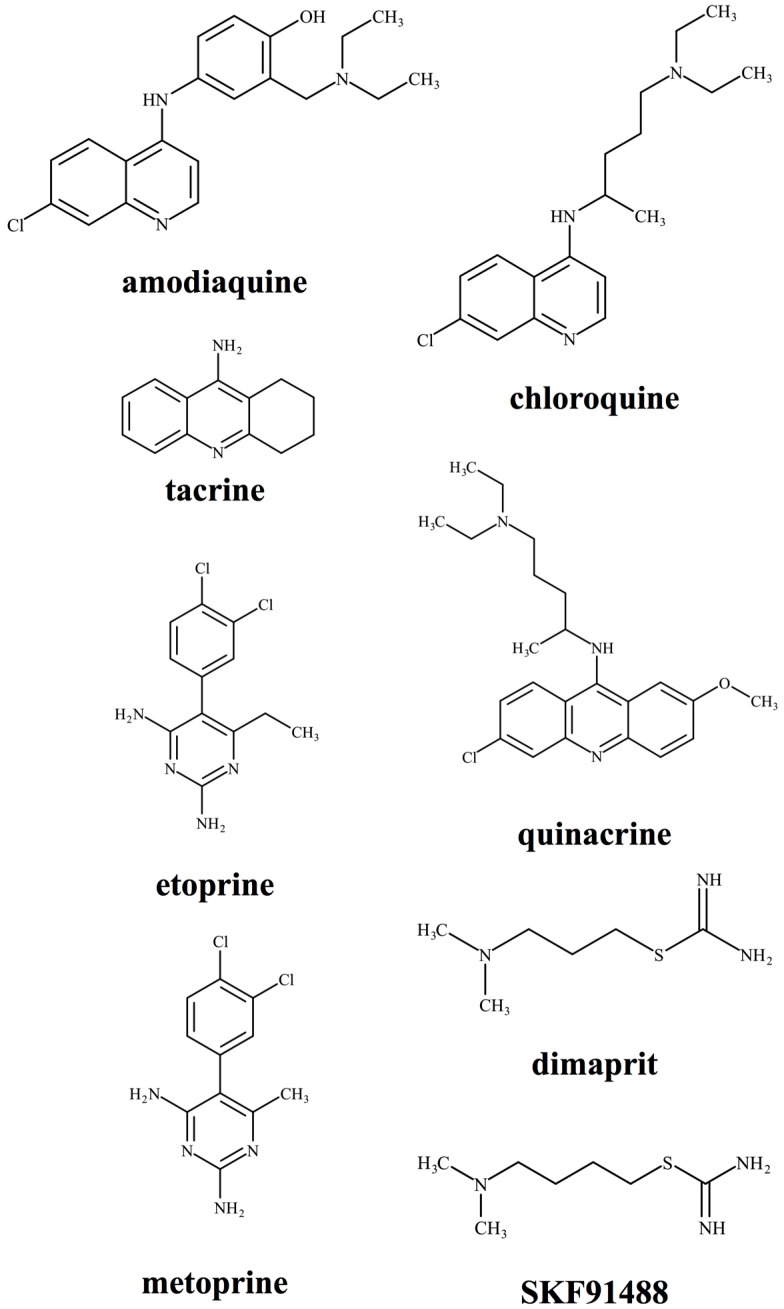
Structures of HNMT inhibitors. Structures of typical HNMT inhibitors amodiaquine, tacrine, etoprine, metoprine, chloroquine, quinacrine, dimaprit, and SKF91488 are depicted.

Duch and colleagues discovered that metoprine, a derivative of 2,4-diaminopyrimidine, inhibited HNMT activity with a Ki value of 100 nM [76,77]. Because metoprine can cross the blood–brain barrier (BBB) due to its hydrophobicity (logP = 2.82) and increase brain histamine concentration [78], many researchers have used this inhibitor to investigate the role of HNMT in the CNS. Pharmacological studies using metoprine indicated that the activation of the histaminergic system in the CNS affects a wide variety of brain functions such as antinociception [79], suppression of energy intake [80], hyperglycaemic action [81], improvement of cognitive function [82], antiepileptic effect [83,84,85], and attenuation of methamphetamine-induced behavioural abnormalities [86]. However, metoprine can also inhibit mammalian dihydrofolate reductase (EC 1.5.1.3) and decrease cellular folate metabolism, resulting in the attenuation of cell growth [87,88]; therefore, it cannot be ruled out that the low specificity of metoprine affected the results. Another HNMT inhibitor, SKF91488, developed by Beaven et al. can specifically inhibit the enzymatic activity devoid of histamine receptor agonist activity [89]. Unfortunately, due to the poor BBB permeability of SKF91488, the brain research using SKF91488 has been limited [90,91,92]. Therefore, novel HNMT inhibitors with high specificity and sufficient BBB permeability are expected to accelerate to pharmacological research on brain HNMT. Properties of commercially available HNMT inhibitors are summarised in Table 3 and Figure 3.

## 5. Phenotyping of *Hnmt*-deficient Mice 

Recently, we phenotyped *Hnmt*-knockout (KO) mice for a better understanding of the role played by HNMT in brain function [31]. First, we showed that histamine content from the cortices, diencephalons, brainstems, and cerebella of KO mice was at least 5-fold higher than that of wild-type (WT) mice. Histamine concentration was consistently higher in whole brain homogenates from neonatal, adolescent, and adult KO mice compared with those of WT mice. Higher extraneuronal histamine concentration in KO mice was also confirmed by in vivo microdialysis assay. However, other monoamines and their metabolites were not changed in KO mouse brains. Histamine concentration in several organs such as the skin and stomach was not elevated. These results demonstrated that Hnmt affected extracellular and intracellular histamine concentration of mouse brains throughout development and adult life. Next, we performed comprehensive behavioural testing to investigate the impact of elevated histamine on mouse behaviours. Although KO mice did not show anxiety-like behaviours, depression-like behaviours, impaired memory, or impaired motor function, highly aggressive behaviours and decreased locomotor activity in home cages were observed in KO mice. High aggression in KO mice was attenuated by pre-treatment of H_2_R antagonist zolantidine but not by H_1_R antagonist pyrilamine, indicating that elevated histamine by *Hnmt* deficiency activated H_2_R, thereby driving aggressive behaviours in mice. The decreased locomotor activity in home cages suggested that the sleep–wake cycle was disrupted in KO mice, resulting in extended immobility during periods that should have been active. Sleep analysis with electroencephalography and electromyography showed prolonged wakefulness during the inactive period and compensatory sleep during the active period. The average wake bout duration was increased in KO mice during zeitgeber time (ZT) 0–6 compared to WT mice, although the total number of wake and sleep bouts was not changed by *Hnmt* deficiency. Pyrilamine injection at ZT0 normalised both the prolonged wakefulness during ZT0–6 and the decreased wakefulness during ZT12–18 in KO mice, indicating that excessive H_1_R activation disrupted the sleep–wake cycle in KO mice. These results demonstrated that HNMT played an essential role in regulating brain histamine concentration and accordingly controls aggression and sleep–wake cycles. In this study, we deleted whole body *Hnmt* to understand the histaminergic nervous system using conventional knockout technology. We could not rule out the possibility that alteration of peripheral organ function might have affected brain functions. Thus, focal disruption of *Hnmt* in adult mice using an adeno-associated virus vector should be utilised to determine brain regions responsible for the aggression and sleep–wake abnormalities. It is also of interest to examine the behavioural changes of KO mice under stressful conditions such as sleep deprivation and social defeat stress so that better understanding of the role of HNMT in psychiatric diseases can be gained. Moreover, the role of Hnmt in rodent models of neurodegenerative disorders including PD and AD should be examined because several clinical studies showed alteration of the histaminergic nervous system in these disorders. Further studies are essential to confirm and extend our findings in rodent models and, eventually, in humans.

## 6. Future Perspectives

As mentioned above, dysfunction of the histaminergic nervous system is associated with various neuropsychiatric disorders including narcolepsy, AD, Tourette’s syndrome, eating disorders, and depression. Animal experiments using H_3_R inverse agonists or HNMT inhibitors suggest the therapeutic effect of histamine elevation on brain function [96]. Dietary intake of histidine, as a precursor of histamine, also improved memory functions via the histamine system in rodents (unpublished observation) and ameliorated feelings of fatigue in humans [97]. The marked effect of HNMT on brain histamine concentration indicates the strong therapeutic potential of HNMT inhibitors against brain diseases. *Hnmt* disruption enhanced brain histamine levels by >6-fold [31], whereas the impact of H_3_R antagonists on brain histamine was quite weak (<2-fold) [98]. Injection of a H_3_R inverse agonist ciproxifan transiently increased histamine release in mouse brains; however, the reduced histamine release was observed 24 h after the injection, possibly due to the development of receptor hypersensitivity or endogenous histamine deletion [99]. Theoretically, HNMT inhibitors do not induce histamine depletion. These results indicate that HNMT inhibitors might have some advantages over H_3_R inverse agonists. Nevertheless, HNMT inhibitors might increase peripheral tissue histamine levels and exacerbate histamine-related diseases such as allergic rhinitis, urticaria, and gastric ulcers, as HNMT is also expressed in peripheral tissues. Several reports have shown that C314T polymorphisms of the *HNMT* gene is not associated with allergic asthma and rhinitis [100,101,102], although a case-control study in Poland indicated the association of this polymorphism with asthma [103]. We confirmed that *Hnmt* disruption did not affect histamine concentration of the skin and stomach [31]. Thus, adverse effects induced by HNMT inhibition in the peripheral tissues might be negligible, although we could not rule out the possibility that HNMT inhibitors might aggravate allergic rhinitis, skin diseases, and peptic ulcers. Taken together, it is intriguing to find novel HNMT inhibitors with high specificity and sufficient BBB permeability. We hope that prominent HNMT inhibitors will help many patients who suffer from brain diseases.

## Figures and Tables

**Table 1:** Basic data of human, mouse, rat, and guinea pig histamine *N*-methyltransferase (HNMT).

		Human [39,48]	Mouse [49]	Rat [50]	Guinea pig [51]
Cloning year		1994	2001	1992	2001
Chromosome		2q22.1	2A3	3p13	N.D.
Amino acid		292 aa	295 aa	292 aa	295 aa
Homology *			83%	83%	81%
K_m_ (µM)	Histamine	13–20	5.3	7.1	N.D.
	SAM	2.0–6.2	5.8	6.3	N.D.

* Homology to human HNMT protein; aa—amino acids; K_m_—Michaelis constant; N.D.—not determined; SAM—S-adenosyl-L-methionine.

**Table 2:** Human *HNMT* mutations and brain diseases.

Authors	Year	SNP	Enzymatic Activity	Disease	Association	Note
Jimenez-Jimenez et al. [58]	2016	C314T (Thr105Ile) (rs11558538) T allele carrier	Decreased	Parkinson’s disease (PD)	Diagnostic OR 0.61	Caucasians and Asians
Yang et al. [57]	2015	C314T (Thr105Ile) (rs11558538) CT hetero allele	Decreased	PD	OR 0.53	Han Chinese
Palada et al. [56]	2012	C314T (Thr105Ile) (rs11558538)	Decreased	PD	Thr105 frequency was associated with PD	Caucasians
Yang et al. [57]	2015	C314T (Thr105Ile) (rs11558538) CT hetero allele	Decreased	Schizophrenia	OR 0.499	Han Chinese
Stevenson et al. [59]	2010	C314T (Thr105Ile) (rs11558538) T allele	Decreased	ADHD	Decreased hyperactivity	Food additives stimulation
Stevenson et al. [59]	2010	A939G (3′-UTR) (rs1050891) G allele	Increased (mRNA stability)	ADHD	Decreased hyperactivity	Food additives stimulation
Meza-Velazquez et al. [60]	2017	C314T (Thr105Ile) (rs11558538) CT hetero allele	Decreased	Migraine	OR 37.10	Migraine-related disability Grade IV
Kellermayer et al. [68]	2017	A939G (3′-UTR) (rs1050891) G allele	Increased (mRNA stability)	Myasthenia gravis (MG)	OR 0.52	Anti-Titin positive MG
Heidari et al. [69]	2015	G179A (Gly60Asp) (rs758252808)	Decreased	Intellectual disability (AR)	Low IQ	Turkish
Heidari et al. [69]	2015	T632C (Leu208Pro) (rs745756308)	Decreased	Intellectual disability (AR)	Low IQ	Kurdish
Marasovic-Susnjara et al. [61]	2011	C314T (Thr105Ile) (rs11558538)	Decreased	Alzheimer’s disease	No association	
Chen et al. [62]	2018	C314 (Thr105Ile) (rs11558538)	Decreased	Amyotrophic Lateral Sclerosis	No association	
Reuter et al. [65]	2007	C314T (Thr105Ile) (rs11558538)	Decreased	Alcoholism	No association	German Caucasians
Oroszi et al. [66]	2006	C314T (Thr105Ile) (rs11558538)	Decreased	Alcoholism	Thr105 frequency was associated with alcoholism.	Finnish Caucasians, Plains Indians
Gracia-Martin et al. [63]	2010	C314T (Thr105Ile) (rs11558538)	Decreased	Multiple sclerosis	No association	
Jimenez-Jimenez et al. [64]	2016	C314T (Thr105Ile) (rs11558538)	Decreased	Restless legs syndrome (RLS)	No association (TT allele might be a risk factor for early onset of RLS)	

PD–Parkinson’s disease; OR–Odds ratio; ADHD–Attention deficit hyperactivity disorder; IQ–Intelligence quotient; SNP—single nucleotide polymorphism.

**Table 3:** Characteristics of HNMT inhibitors.

Inhibitors	M.W.	IC_50_ or K_i_	Inhibition Pattern	Note
Amodiaquine	355.86	K_i_ 18.6 nM (recombinant hHNMT) [93] IC_50_ 400 nM (recombinant hHNMT) [39]	Mixed	An antimalarial drug
Chloroquine	319.88	IC_50_ 600 nM (guinea pig skin) [94] IC_50_ 12.6, 22.0 19.0 and 21.7 µM (human liver, renal cortex, brain and colon) [44]	Competitive to histamine	An antimalarial drug
Dimaprit	161.27	K_i_ 8 µM (rat kidney) [89] Ki 7–9 µM (guinea pig brain) [89]	Noncompetitive to histamine	H_2_R agonist
Etoprine	283.16	K_i_ 760 nM (rat brain) [76]	N.D.	Dihydrofolate reductase inhibitor
Metoprine	269.13	K_i_ 100 nM (rat brain) [76] K_i_ 91 nM (recombinant hHNMT) [93]	Competitive to histamine	Dihydrofolate reductase inhibitor
Quinacrine	399.96	IC_50_ 160 nM (guinea pig skin) [94] K_i_ 450 nM (recombinant hHNMT) [52]	Competitive to histamine	An antimalarial drug
SKF91488	175.29	K_i_ 0.9–1.6 µM (rat kidney) [89] K_i_ 1.85 µM (recombinant rat HNMT) [50] K_i_ 3 µM (guinea pig brain) [89]	Noncompetitive to histamine	Poor BBB permeability
Tacrine	198.27	K_i_ 38.2 nM (recombinant hHNMT) [93] K_i_ 35 nM (rat kidney) [95]	Competitive to histamine	Acetylcholinesterase inhibitor

IC_50_—half maximal inhibitory concentration; K_i_—inhibitory constant; M.W.—molecular weight; N.D.—not determined.
